# Comparison of beta diversity measures in clustering the high-dimensional microbial data

**DOI:** 10.1371/journal.pone.0246893

**Published:** 2021-02-18

**Authors:** Biyuan Chen, Xueyi He, Bangquan Pan, Xiaobing Zou, Na You

**Affiliations:** 1 Child Development and Behavior Center, The Third Affiliated Hospital, Sun Yat-sen University, Guangzhou, China; 2 School of Mathematics, Sun Yat-sen University, Guangzhou, China; University of Hong Kong, HONG KONG

## Abstract

The heterogeneity of disease is a major concern in medical research and is commonly characterized as subtypes with different pathogeneses exhibiting distinct prognoses and treatment effects. The classification of a population into homogeneous subgroups is challenging, especially for complex diseases. Recent studies show that gut microbiome compositions play a vital role in disease development, and it is of great interest to cluster patients according to their microbial profiles. There are a variety of beta diversity measures to quantify the dissimilarity between the compositions of different samples for clustering. However, using different beta diversity measures results in different clusters, and it is difficult to make a choice among them. Considering microbial compositions from 16S rRNA sequencing, which are presented as a high-dimensional vector with a large proportion of extremely small or even zero-valued elements, we set up three simulation experiments to mimic the microbial compositional data and evaluate the performance of different beta diversity measures in clustering. It is shown that the Kullback-Leibler divergence-based beta diversity, including the Jensen-Shannon divergence and its square root, and the hypersphere-based beta diversity, including the Bhattacharyya and Hellinger, can capture compositional changes in low-abundance elements more efficiently and can work stably. Their performance on two real datasets demonstrates the validity of the simulation experiments.

## Introduction

The heterogeneity of disease is the primary concern of precision medicine, and it challenges medical research in many aspects, from the identification of risk factors to the development of specific treatments [1–3]. Patients with the same perceived disease may respond quite differently to the same treatment and show distinct prognoses in clinical practice. Most common diseases are so complex that they have various subtypes, and the etiology and pathogenesis of patients vary between subtypes [3–5]. Rather than treating patients uniformly, it is more reasonable to classify them into subgroups and develop different specific treatments for different subgroups.

Recently, many studies have indicated that the gut microbiome plays an important role in the origin and development of disease through the gut-brain axis [6–9]. Because of the advantages of high efficiency and low cost, the abundance of microbial genes in gut samples is popularly measured by 16S rRNA high-throughput sequencing [10]. The analysis pipeline [11] classifies the sequenced reads into operational taxonomic units (OTUs) and measures their abundance by the binned read coverage. Annotating the OTU sequences at different taxonomic levels yields the microbial compositions and their abundance at different levels. Considering the microbial evolution and taxonomy accuracy of 16S rRNA sequencing, the analysis at the genus level is of great interest, where the OTU abundance of each sample is represented as a high-dimensional vector. Through advances in sequencing technology, we can detect the large-scale microbiome inside human bodies. Their abundance varies within a vast range, from couples to millions. After normalization to make the total composition of each sample one, a large proportion of extremely small values appears in the vector; many zeros may even be included when the compositional data of different samples are summarized in an OTU table.

The clustering of microbial samples based on their compositions reveals the heterogeneity of patients in terms of the gut microbiome. The clustered subgroups are characterized as enterotypes, which attract researchers’ attention when they appear [12–14]. To classify the samples into subgroups according to their compositional profiles, the dissimilarity between samples needs to be measured, which is termed beta diversity in the microbial community. The definition of beta diversity was first introduced by ecologists, together with alpha and gamma diversity, to measure the diversity between samples, within samples and of the total population [15, 16]. Since then, many different types of definitions of beta diversity have emerged from different perspectives [17]. Because the aim is to quantify the dissimilarity between two compositional vectors, mathematical metrics that measure the difference between two multivariate variables can be employed and can provide a variety of definitions of beta diversity with different conceptual and sampling properties [18]. The R package phyloseq [19] includes 41 such measures, and philentropy [20] covers 46. These include not only the commonly used Euclidean and Jensen-Shannon divergence but also diversity measures for presence-absence data [21] as well as the UniFrac distance utilizing phylogenetic information [22]. Recently, scientists have made efforts to refine the definition of beta diversity in both mathematical and conceptual terms [23]. Although there are fruitful choices for beta diversity and valuable discussions on their concepts, different measures may yield significantly different clusters in practical data analysis [24]. It is confusing for users to make one selection from the clusters resulted from different beta diversity measures, even with indices such as the Caliński-Harabasz statistic, silhouette coefficient, and prediction strength, to evaluate the clustering performance, since different indices may give different recommendations [13, 24].

Numerical evaluation based on simulations can provide an objective comparison of the performance of different beta diversity measures. However, previous works have mainly focused on the analysis of low-dimensional data [25–27]. In this paper, we set up three simulation experiments to mimic microbial compositions in order to investigate the performance of different beta diversity measures in clustering high-dimensional compositional data. By comparison with the truth, we can infer in what situations the beta diversity can have better performance in order to guide the choice of beta diversity in practical data analysis. Note that in this study, we focus on the measures defined in terms of the abundance rather than the presence-absence data. Presence-absence data may be more sensitive to rare compositions. However, it is risky to consider only the presence or absence of high-dimensional microbial data with many extremely small compositional elements, since OTUs at extremely low abundance may appear, possibly due to sequencing errors or annotation errors. Neither UniFrac nor weighted UniFrac are considered in this comparison analysis because we simulate and make inferences based on the OTU table, which does not carry phylogenetic tree information.

We choose 13 beta diversity measures that are popularly used in microbial studies and compare their performance in clustering high-dimensional compositional data. The paper is structured as follows: In the Methods section, we present the definition of each type of beta diversity under investigation. Three simulation experiments are introduced in the Results section to evaluate the clustering performance of the different beta diversity measures. The analysis of two real datasets is subsequently given. A Discussion section is presented at the end.

## Methods

Denoting by ***x***_*i*_ = (*x*_*i*1_, ⋯, *x*_*im*_)′ the *m* compositions of the *i*th subject in the population ***x***_1_, …, ***x***_*N*_, the compositional constraints *x*_*ik*_ ≥ 0 and $\sum_{k=1}^{m}x_{ik}=1$ hold for *i* = 1, 2, …, *N*. Given a prespecified number of clusters *G*, a clustering algorithm, such as the partitioning around medoids method (PAM) [28], is used to classify the population into *G* groups according to the dissimilarity matrix *D* = (*d*_*ij*_)_*N*×*N*_, where *d*_*ij*_, termed the beta diversity, quantifies the dissimilarity between the compositional vectors of two distinct samples ***x***_*i*_ and ***x***_*j*_. Based on different dissimilarity measures, beta diversity can be defined by different formulations, as listed in Table 1.

**Table 1:** Definitions of different beta diversity measures.

Category	Notation	Name	Expression
Euclidean-based measures	*β*_1_	Euclidean	$\sqrt{\sum_{k=1}^{m}(x_{ik}-x_{jk}{)}^{2}}$
	*β*_8_	Angular	$\text{arccos}(\frac{\sum_{k=1}^{m}x_{ik}x_{jk}}{\sqrt{\sum_{k=1}^{m}x_{ik}^{2}}\sqrt{\sum_{k=1}^{m}x_{jk}^{2}}})$
	*β*_9_	Horn-Morisita	$1-\frac{2\sum_{k=1}^{m}x_{ik}x_{jk}}{\sum_{k=1}^{m}x_{ik}^{2}+\sum_{k=1}^{m}x_{jk}^{2}}$
Manhattan-based measures	*β*_2_	Manhattan	$\sum_{k=1}^{m}|x_{ik}-x_{jk}|$
	*β*_3_	Bray-Curtis	$\frac{\sum_{k=1}^{m}|x_{ik}-x_{jk}|}{\sum_{k=1}^{m}(x_{ik}+x_{jk})}$
	*β*_4_	Jaccard	$1-\frac{\sum_{k=1}^{m}\text{min}(x_{ik},x_{jk})}{\sum_{k=1}^{m}\text{max}(x_{ik},x_{jk})}$
KL-based measures	*β*_5_	J-divergence	$\sqrt{D(x_{i}\|x_{j})+D(x_{j}\|x_{i})}$
	*β*_6_	JSD	$\frac{1}{2}[D(x_{i}\|\frac{x_{i}+x_{j}}{2})+D(x_{j}\|\frac{x_{i}+x_{j}}{2})]$
	*β*_7_	rJSD	$\sqrt{\frac{1}{2}[D(x_{i}\|\frac{x_{i}+x_{j}}{2})+D(x_{j}\|\frac{x_{i}+x_{j}}{2})]}$
hypersphere-based measures	*β*_10_	Bhattacharyya	$\text{arccos}(\sum_{k=1}^{m}\sqrt{x_{ik}}\sqrt{x_{jk}})$
	*β*_11_	Hellinger	$\sqrt{\sum_{k=1}^{m}(\sqrt{x_{ik}}-\sqrt{x_{jk}}{)}^{2}}$
Aitchison-based measures	*β*_12_	Manhattan-ilr	$\sum_{k=1}^{m}|r_{ik}-r_{jk}|$
	*β*_13_	Euclidean-ilr	$\sqrt{\sum_{k=1}^{m}(r_{ik}-r_{jk}{)}^{2}}$

$D(x_{i}\|x_{j})=\sum_{k=1}^{m}x_{ik}\text{ln}(x_{ik}/x_{jk})$ indicates the Kullback-Leibler divergence.

$\lambda_{x_{i}}=\sum_{k=1}^{m}x_{ik}^{2}/(\sum_{k=1}^{m}x_{ik}{)}^{2}=\sum_{k=1}^{m}x_{ik}^{2}$.

***r***_***i***_ = (*r*_*i*1_, ⋯, *r*_*im*_)′ = *ilr*(***x***_***i***_).

The most commonly used metrics, Euclidean *β*_1_ and Manhattan *β*_2_ [20], are actually the *L*_2_ or *L*_1_ norm developed in real space. The Bray-Curtis *β*_3_ [19], also called Canberra [20], metric gives a 1/2 multiplied dissimilarity matrix of Manhattan since *β*_3_ = *β*_2_/2 due to $\sum_{k=1}^{m}x_{ik}=\sum_{k=1}^{m}x_{jk}=1$. It yields the same clustering result as Manhattan and will not be calculated in the comparison analysis. The Jaccard *β*_4_ [19], or Tanimoto [20], metric is a monotone function of Manhattan *β*_2_, i.e., *β*_4_ = 2*β*_2_/(2 + *β*_2_). Due to these close relationships between Manhattan, Bray-Curtis and Jaccard, we denote them as Manhattan-based measures in Table 1.

The Kullback-Leibler (KL) divergence [29] reflects the difference between two probability measures. Its discrete version can be directly applied to measure the dispersion between two compositional vectors, yielding the J-divergence *β*_5_ [27] and the widely used Jensen-Shannon divergence (JSD) [20] in Table 1. The JSD does not satisfy the triangle inequality and is not a mathematical distance, but its square root, rJSD $\beta_{7}=\sqrt{\beta_{6}}$ [30], is, so rJSD is usually alternatively employed in the literature on enterotype studies [12, 13]. These three beta diversities are referred to as KL-based measures in Table 1. According to the expression of J-divergence $\beta_{5}=\sqrt{\sum (x_{ik}-x_{jk})(\text{ln}\;x_{ik}-\text{ln}\;x_{jk})}$, it measures not only the absolute difference between two compositions but also those with log transformations. Since the compositions are restricted to small values between 0 and 1, the incorporation of the logarithm may offer J-divergence more power in quantifying compositional changes compared to the measures developed at the original scale, such as Euclidean, Manhattan and Jaccard. Both the JSD and rJSD also acquire this advantage by utilizing the logarithm through the KL divergence. In contrast to the J-divergence, the JSD and rJSD use (***x***_*i*_ + *x*_*j*_)/2 instead of ***x***_*i*_ and ***x***_*j*_ themselves as the reference distribution in the calculation of the KL divergence. This strategy makes them slightly less sensitive to small differences between ***x***_*i*_ and ***x***_*j*_ compared with the J-divergence. In data analysis, compositional changes in different elements are presented at varying magnitudes. Emphasizing the smaller changes may be either helpful or harmful for clustering, and this depends on the relative magnitude of the between-cluster signals and the within-cluster noises, as shown later in the simulations. Based on the formulas in Table 1, the J-divergence does not allow zero compositions in *x*_*ik*_ or *x*_*jk*_, and neither do the JSD or rJSD when both of them are zero. In our analysis, we use the R package philentropy [20] for computation, where *x*/0 is replaced by *x*/*ϵ* and *x*ln(0) by *x*ln(*ϵ*) and *ϵ* = 1e-5.

The compositional vectors of *m* dimensions vary within the *m* − 1 dimensional simplex space [25]; for instance, when *m* = 3, the vectors are a triangle formed by three vertexes, (1, 0, 0)′, (0, 1, 0)′ and (0, 0, 1)′, with its interior. Considering the limiting variation of compositional vectors in the radii, the angle contained by two vectors with a center at **0**, Angular *β*_8_ [27], reflects the dispersion between their compositions to a great extent. Note that Euclidean *β*_1_ is the chord length between two compositional vectors corresponding to the angle *β*_8_. In addition, Horn-Morisita *β*_9_ [19], abbreviated to Horn, which is also called Dice of Drost [20], is related to Angular by $\beta_{9}=1-2\;\text{cos}\;\beta_{8}\sqrt{\sum_{k}x_{ik}^{2}}\sqrt{\sum_{k}x_{jk}^{2}}/(\sum_{k}x_{ik}^{2}+\sum_{k}x_{jk}^{2})$ and Euclidean via $\beta_{9}=\beta_{1}^{2}/(\sum_{k}x_{ik}^{2}+\sum_{k}x_{jk}^{2})$. These connections may make them have similar clustering results. We denote them as Euclidean-based measures in Table 1. According to their formulas, these measures differ from each other when the variances of the within-sample compositions $\sum_{k}x_{ik}^{2}$ and $\sum_{k}x_{jk}^{2}$ vary, whereas $\sum_{k}x_{ik}^{2}$ and $\sum_{k}x_{jk}^{2}$ are the squared radii of the compared compositional vectors.

It is arbitrary to take the radius out of consideration or account for it in certain manners as Horn does in measuring the dissimilarity in the simplex space. The mapping from ***x***_*i*_ to $\sqrt{x_{i}}$, *i* = 1, 2, …, *N*, yields a projection of the simplex space onto a unit hypersphere with the same radius and derives the beta diversity measures defined by the angle in the hypersphere space, named *β*_10_ by Bhattacharyya [27], and the chord length, the Hellinger *β*_11_ [27]. They are more reasonable in dealing with the effect of the radii than Angular and Horn, referred to as hypersphere-based measures in Table 1. In addition, square-root mapping leads them to favor the differentials between compositions at a low abundance.

The log transformations proposed by Aitchison [25] set up the foundations for composition modelling, where *alr*(***x***_*i*_) = (ln(*x*_*i*1_/*x*_*im*_), …, ln(*x*_*i*,*m*−1_/*x*_*im*_))′ maps the *m*-dimensional simplex space *S*^*m*^ to the (*m* − 1)-dimensional real space *R*^*m*−1^; *clr*(***x***_*i*_) = (ln(*x*_*i*1_/*g*(***x***_*i*_)), …, ln(*x*_*im*_/*g*(***x***_*i*_)))′ with $g(x_{i})=(\prod_{k=1}^{m}x_{ik}{)}^{1/m}$ converts *S*^*m*^ to a hyperplane in real space *U*^*m*^ = {(*u*_1_, …, *u*_*m*_): *u*_1_ + … + *u*_*m*_ = 0}; and *ilr*(***x***) = *V*′*clr*(***x***) projects *S*^*m*^ to *R*^*m*−1^, where *V*′ is the transport of the *m* × (*m* − 1) matrix *V*, whose columns form an orthonormal basis of *U*^*m*^ [31]. The dissimilarity measures developed in real space, such as Euclidean and Manhatten, can be applied to the transformed data and serve as the beta diversity for compositional vectors. We note that none of these three transformations is compatible with zero compositions. The R package compositions [32] calculates *clr* and *ilr* by omitting zeros for the transformation and then patching them back in. Considering the close relationship between *clr* and *ilr*, we use only *ilr* for data transformation and then apply the Manhattan and Euclidean distance to calculate the beta diversity on the transformed data; these are denoted as Aitchison-based measures in Table 1.

## Results

### Simulations

To investigate the performance of different beta diversity measures in clustering the population into subgroups, we set up three simulation experiments to mimic the microbial compositional data. Throughout the simulations, we set *m* = 500 and *G* = 2 clusters, each with *N* = 100 samples. Using each type of beta diversity presented in Table 1, we obtain a distance matrix and then apply the PAM for clustering analysis. The adjusted Rand index (ARI) [33] is used for the assessment of the clustering accuracy. Each experiment is repeated 500 times, and the average ARI is calculated for the evaluation.

#### Experiment 1

In the first experiment, we generate the compositional vectors using the log-normal distribution, as stated in Lu et al. [34]. Denoting by *LN*(***μ***, Σ) the multivariate log-normal distribution with mean ***μ*** and covariance matrix Σ, the random vector ***z*** = (*z*_1_, …, *z*_*m*_)′, which is generated from *LN*(***μ***, Σ), is converted to a compositional vector via $x=z/\sum_{i=1}^{m}z_{i}$. We set ***μ*** = *μ*_*g*_ in cluster *g*, *g* = 1, 2, and Σ = (0.5^|*i*−*j*|^)_*m*×*m*_ is the same in both clusters. The elements in ***μ***_1_ are assigned randomly using the normal distribution *N*(***μ***, *σ*) with mean *μ* and standard deviation *σ*, and ***μ***_2_ is constructed by manipulating ***μ***_1_. Specifically, the first 50 elements (10% of the total) of ***μ***_1_ are generated independently from *N*(9, 1), the next 50 (10%) from *N*(6, 1) and the remaning 400 (80%) from *N*(3, 1), resulting in compositions of cluster 1 at three levels of abundance, which are high at approximately 1e-2, median at approximately 4e-4 and low at approximately 2e-5. To explore how the compositional changes affect the clustering results using different beta diversity measures, we randomly select 10% of the ***μ***_1_ elements at different abundance levels and add perturbations to construct ***μ***_2_. Perturbing the high-level ***μ***_1_ elements by *N*(0, 1), the median by *N*(0, 3) and the low level by *N*(0, 5), we obtain three simulation scenarios, 1.1, 1.2 and 1.3.

**Fig 1:**
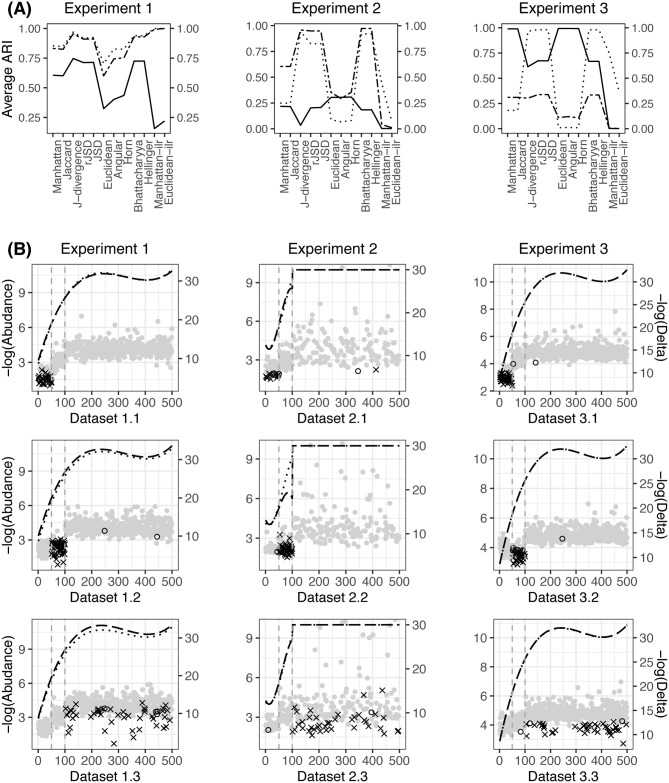
**(A)**: The average ARIs obtained in simulation experiments 1-3, where the solid, dashed and dotted lines indicate scenarios x.1-x.3, respectively. **(B)**: The dashed and dotted lines represent the cubic smoothing spline of -log(Abundance), where Abundance indicates the average abundance of two clusters along all the elements. The dots represent -log(Delta), where Delta is the absolute mean difference between two clusters along all the elements, × indicates the coordinates with p-values of the Wilcoxon signed-rank test between two clusters that are smaller than 0.001, and ∘ indicates the coordinates with p-values between 0.001 and 0.01.

The average ARIs obtained using different beta diversities in Table 1 are presented in Fig 1(A). It is shown that the measures in the same category perform similarly in terms of clustering and differ from those in the other categories. As stated in Section 2, the measures in the same category have a close relationship, which is why they yield very similar clustering results. Among these beta diversities, the Aitchison-based measures seem unstable, and the average ARIs may be either the best or the worst compared to the others in different scenarios.

The implemented perturbations cause compositional changes between clusters. To investigate how the clusters are separated in the analyzed data, we randomly select a representative dataset from each scenario, indicated as datasets 1.1, 1.2 and 1.3, and illustrate the compositions of each cluster along all the elements in the first column in Fig 1(B). The abundances of the two clusters are very close to each other. To better visualize the compositional difference between each pair of clusters, we present their absolute mean difference along all the elements in Fig 1(B). Note that the significance of the differential between two clusters, rather than the absolute difference value, reveals the between-cluster dispersion and determines the clustering results. We highlight the elements with significant p-values in the Wilcoxon signed-rank test in Fig 1(B). From dataset 1.1 to 1.3, it is shown that the significant between-cluster differences move from the high-abundance to the median-abundance and then to the low-abundance elements, while their corresponding absolute mean differences decrease.

Other than the Aitchison-based measures, the performance of the various beta diversities is determined by their ability to capture different levels of compositional changes between the clusters. As seen in Fig 1(B), all the compositional changes are actually of very small magnitudes. The logarithm implemented by the KL-based measures helps reflect the tiny numerical changes, as does the square root that the hypersphere-based measures utilize, which leads them to achieve higher ARIs than the Manhattan- and Euclidean-based measures. Although measures within the same category perform similarly in many situations, it is notable that they may present quite different ARIs; for instance, the J-divergence gives a higher ARI than the JSD and rJSD, and the Euclidean distance yields a significantly lower ARI than the Angular and Horn. Nevertheless, no matter how the ARIs vary within the categories, the KL- and hypersphere-based measures can always produce the top ARIs compared with the others.

#### Experiment 2

The multivariate Dirichlet distribution is a natural choice to generate compositional vectors. In the second experiment, we simulate the clusters according to the multivariate Dirichlet distribution *D*(***α***), where ***α*** is a positive parameter of length *m* and ***α*** = ***α***_*g*_ in the *g*th cluster, *g* = 1, 2. The first 50 elements(10% of the total) of ***α***_1_ are generated independently from the chi-square distribution *χ*^2^(10) with 10 degrees of freedom, the following 50 (10%) are from *χ*^2^(1) and the remaining 400 (80%) are from *χ*^2^(0.1). These correspond to three levels of abundance in cluster 1, which are high around 2e-2, median around 3e-4, and low; over 85% are less than 1e-10, including zero values. Similar to Experiment 1, ***α***_2_ is set up by manipulating ***α***_1_. The random perturbations of *χ*^2^(2), *χ*^2^(1) or *χ*^2^(1/2) are superposed on 50 high-, median-, or low-abundance elements, resulting in scenarios 2.1, 2.2 and 2.3, respectively. The average ARIs obtained in the three scenarios and three representative datasets 2.1, 2.2 and 2.3 are illustrated in the second column in Fig 1.

The average ARIs of the KL- and hypersphere-based beta diversity are significantly higher than those of the Manhattan- and Euclidean-based beta diversity in scenarios 2.2 and 2.3; however, in scenario 2.1, the former did not show such an advantage. As presented by the representative datasets, the significant differences between the clusters in scenarios 2.1-2.3 are mainly located in high-, median- and low-abundance elements, respectively. Considering the superiority of the KL- and hypersphere-based measures in quantifying the differentials at smaller values, it is not surprising that they present higher ARIs in scenarios 2.2 and 2.3. Unlike the simulated data in experiment 1, many more abundances that are extremely low were generated in this experiment. When the significant between-cluster differences are located in those elements, using KL- or hypersphere-based beta diversity would improve the clustering. However, if the significant differences are not located in them but some higher abundance such as in scenario 2.1, the KL- or hypersphere-based measures will magnify the noises from those extremely low-abundance elements as well, and this may degrade the clustering. The vanishing superiority of ARIs using the KL- and hypersphere-based measures in scenario 2.1 confirms this finding. The Aitchison-based measures do not show competitive ARIs in any scenario of this experiment.

#### Experiment 3

It is worth noting that the perturbations in the parameter ***μ*** of the log-normal distribution or ***α*** of the Dirichlet distribution have no straightforward relationship with the compositional changes in ***x***_*i*_. Due to the correlations within the sample compositions, parameter perturbations at one level may also bring compositional changes at the other levels. To minimize this impact on the conclusions, we set up a third experiment to simulate the datasets using the multinomial distribution *Mul*(*N*, ***P***), where *N* is the total count, and ***P*** = (*P*_1_, …, *P*_*m*_)′, where *P*_*i*_ ≥ 0 and $\sum_{i=1}^{m}P_{i}=1$.

First, we estimate ***P*** and the distribution of *N* by the Monte Carlo method. A total of 10,000 compositional vector replicates are generated according to the simulation settings of cluster 1 in the first experiment, representing an empirical distribution ${\hat{F}}_{N}$ of *N*, and a Monte Carlo estimate $\hat{P}$ for ***P***, the first 10% of the elements of which are at high abundance around 1e-2, followed by 10% median around 8e-4, and 80% low around 3e-5. Then, we let $P_{1}=\hat{P}$ and generate the compositions in cluster 1 in three steps, first generating *N* from ${\hat{F}}_{N}$, then simulating the vector of counts from *Mul*(*N*, ***P***_1_), and finally normalizing the counts as compositions by dividing them by their summation. A subset of *s* elements in ***P***_1_, denoted by ***Q***_*s*_, is collected and perturbed as $Q_{s}^{\prime }=Q_{s}⊕\epsilon$, where ⊕ is the addition operator in the simplex space [25] and *ϵ* is a random sample from *D*(*γ* ⋅ **1**). Therefore, ***P***_2_ is obtained by replacing ***Q***_*s*_ in ***P***_1_ with $Q_{s}^{\prime }$ and is used to generate cluster 2. We randomly select *s* = 50 elements from those at high abundance for the perturbation *γ* = 10, 000, median for *γ* = 1, 000, and low for *γ* = 10, resulting in scenarios 3.1, 3.2 and 3.3, respectively. The average ARIs and three representative datasets 3.1, 3.2 and 3.3 are presented in the last column of Fig 1.

Similar to Experiment 2, when the compositional changes are intended to be at a high abundance level in scenario 3.1, the KL- and hypersphere-based beta diversity may yield smaller ARIs than the Manhattan- and Euclidean-based measures. As the compositional changes move to lower levels in scenarios 3.2 and 3.3, the advantage of the KL- and hypersphere-based measures becomes increasingly significant. In addtion, in scenarios 3.1 and 3.2, the J-divergence provides smaller ARIs than the JSD and rJSD, as in scenario 2.1. Corresponding to the discussion in Section 2 based on their definitions, these numerical results demonstrate that the J-divergence entails a greater risk that the between-cluster signals are obscured by the within-cluster variations from the lower abundance levels. Considering the highest ARIs from the J-divergence in Experiment 1, it is implied that the J-divergence is more data-dependent than the JSD and rJSD.

#### Conclusion

In addition to the Aitchison transformations, which are not applicable to the high-dimensional compositional data analysis and show fluctuating results, the beta diversity measures under investigation in this paper can be partitioned into two classes, the Manhattan- or Euclidean-based measures and the KL- or hypersphere-based measures. Measures belonging to the same class have similar clustering results. Comparatively, the former emphasizes compositional changes at higher-abundance elements, while the latter favors differentials at a lower abundance. Therefore, to cluster the high-dimensional compositional data, if the diversity at high abundance is of interest, the measures in the former class are suggested. Among them, the J-divergence is given the lowest priority due to its data dependency. Meanwhile, if the dispersion of rare compositions is involved, then the measures in the latter class are recommended.

### Real analysis

#### Autism dataset

This is the dataset that motivated this study. The gut samples of 278 children were collected by the Third Affiliated Hospital of Sun Yat-sen University to explore the microbial biomarkers for autism, including 209 autism patients and 69 healthy controls. Their enterotypes are of primary interest, and we became aware of the difference between the clustering results using different beta diversity measures during the exploration. We preprocessed their 16S rRNA sequencing data according to the QIIME pipeline [35]. The microbial genome annotation at the genus level yielded the compositions of 278 samples among 780 OTUs, which are summarized in a data matrix and available in Table in S1 Table. The 50%, 75%, 90%, and 95% quantiles of the compositional values are 5.2e-7, 4.4e-6, 6.2e-5, and 8.3e-4, respectively. In particular, 87.5% of the elements of the OTU table are zeros, while only 1.7% are higher than 0.01 and 3.5% are greater than 0.001. We used the beta diversity in Table 1 to cluster the population into *G* = 2 to 10 subgroups and calculated the Caliński-Harabasz indices, silhouette coefficients and prediction strengths of these clustering results. These indices do not significantly increase as *G* changes from 2 to 10. Therefore, we set *G* = 2 in the following clustering analysis.

**Fig 2:**
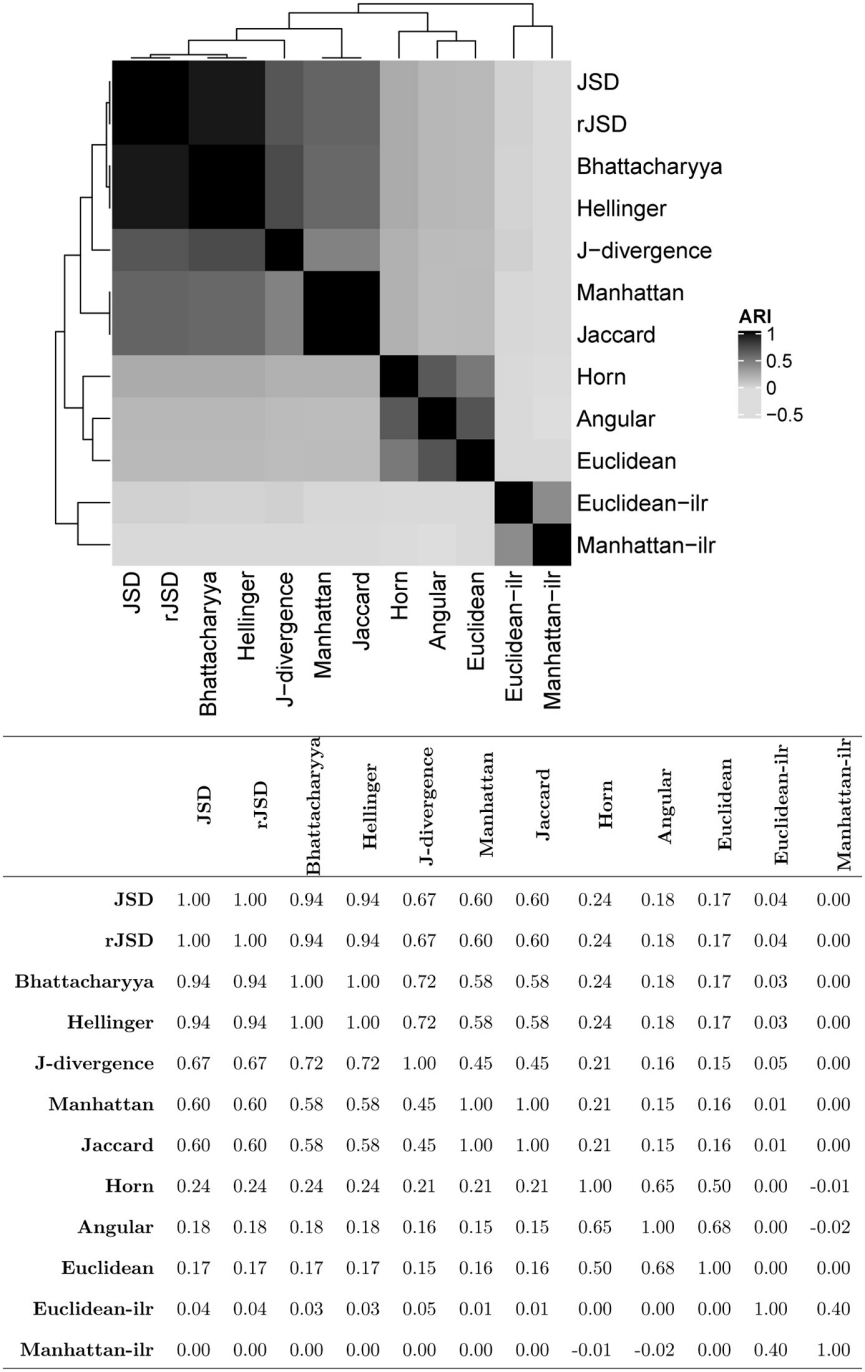
Heatmap and values of the pairwise ARIs of clustering results using different beta diversity measures in the autism dataset.

Using different beta diversities, the samples are rearranged into different clusters. To reflect the variation among the clustering results from the different measures, we calculate their pairwise ARIs and present them as a heatmap in Fig 2. According to the hierarchical tree in the heatmap, the Aitchson-based beta diversity yields significantly different clusters from the others. The KL- and hypersphere-based measures perform similarly and differently from the Manhattan- and Euclidean-based beta diversity. Among the group of KL- and hypersphere-based measures, the J-divergence departs slightly from the others. The classification of the beta diversity measures is consistent with that in the simulation, where two classes that favor compositional changes at low or high abundance are identified. We further investigated the significantly different OTUs between the clusters that were obtained using different beta diversity measures. The numbers of OTUs whose adjusted p-values with false discovery rate (FDR) control are smaller than 0.05 and their mean abundances are listed in Table 2. Except for the Aitchison-based measures, the KL- and hypersphere-based beta diversity yielded the clusters with the most OTUs with adjusted p-values less than 0.05. The additional acquired differential OTUs are mainly located in the elements whose mean abundance is lower than 0.001, demonstrating the superior capability of the KL- and hypersphere-based measures in determining the compositional changes at low abundance levels.

**Table 2:** Numbers of OTUs whose adjusted p-values with FDR control are smaller than 0.05, and their frequencies at different abundance levels.

	Total	OTU mean abundance		
		>0.001	0.001∼1e-5	<1e-5
**Manhattan**	23	14	9	0
**Jaccard**	23	14	9	0
**J-divergence**	29	18	11	0
**JSD**	35	19	15	1
**rJSD**	35	19	15	1
**Euclidean**	4	4	0	0
**Angular**	7	5	1	1
**Horn**	3	3	0	0
**Bhattacharyya**	33	18	15	0
**Hellinger**	33	18	15	0
**Manhattan-ilr**	84	5	42	37
**Euclidean-ilr**	82	16	39	27

#### Human gut metagenomes

Arumugam et al. [12] first proposed the concept of an enterotype by clustering 33 fecal samples using rJSD into three subgroups according to 249 OTUs annotated at the genus level (available online at: https://enterotype.embl.de/MetaHIT_SangerSamples.genus.txt). They defined three enterotypes, which are named *Bacteroides*, *Prevotella*, and *Ruminococcus* and have sample sizes of 19, 6 and 8, respectively. We apply all the beta diversity measures included in this paper to reanalyze the OTU table. The JSD and hypersphere-based measures provide exactly the same clusters as rJSD. The J-divergence yields a unique but quite similar clustering results to rJSD, only moving one sample in *Ruminococcus* to *Bacteroides*, with an ARI between these two clustering results of 0.90. The Manhattan- and Euclidean-based measures also obtain the same partitions. They move two samples from *Prevotella* to *Bacteroides*, with an ARI of 0.82 compared to the clusters from rJSD. The Aitchison-based measures present very distinct clusters from rJSD, and the ARIs of their clustering results with those of rJSD are only 0.02 and 0.15.

The consistency between the clusters from different beta diversity measures indicates that the compositional changes in this dataset may be mainly located at a high abundance level. Compared to rJSD, the rearrangement of FR.AD.3 from *Ruminococcus* to *Bacteroides* by the J-divergence yields more significantly different OTUs between clusters with low abundances, such as *Akkermansia* and *Gordonibacter*, whose highest compositions are 0.09 and 0.003, respectively. Moving two samples, DA.AD.4 and FR.AD.6, from *Prevotella* to *Bacteroides* by the Manhattan- and Euclidean-based measures results in a number of OTUs whose adjusted p-values with FDR control are smaller than 0.1, decreasing from 4 to 2. The compositions of 2 vanishing OTUs, *Rhodospirillum* and *Escherichia/Shigella*, are both at low abundances, less than 2e-5 and 0.035, respectively. It is shown that the rJSD, JSD and hypersphere-based measures focus more on smaller compositional changes at lower abundances than the Manhattan- and Euclidean-based measures, and the J-divergence may further enhance this trend.

## Discussion

In this paper, we propose three simulation experiments to mimic high-dimensional compositional clusters and investigate the performance of different beta diversity measures in clustering compositional samples into subgroups. The conclusions can be used to guide the choice of beta diversity and explain the difference in the resulting clusters using different beta diversity measures.

Through the simulations, we aim to determine how the beta diversity measures perform for different settings of the clusters. The high-dimensional compositions are simulated using common statistical distributions, and ideal clusters with specific levels of compositional changes are generated to simplify the data complexity for easy clarification of the conclusions. We considered only *G* = 2 for convenience in discussion. The findings are general and can be extended to populations with more than two clusters since the dispersion in a more complicated population is composed of compositional changes between any two of the clusters.

In addition to the PAM, there are many other clustering algorithms in statistics, such as K-means and hierarchical clustering [28]. Because of its robustness and easy compatibility with the distance matrices from different beta diversity measures, the PAM is popularly employed for clustering analysis in microbial studies [12, 13], so we chose the PAM to cluster the samples in this analysis. In addition, both the beta diversity and clustering algorithm may affect the clustering results. To eliminate the impact of different choices on the clustering algorithms and focus on the performance of different beta diversity measures, we fixed the use of the PAM in this study.

For real applications, compositional changes may be located at multiple levels of abundance simultaneously, and no single beta diversity measure can capture all the signals. Researchers have to combine the results for comprehensive consideration or choose one according to their needs, i.e., depending on whether the diversity at a high or low level of abundance is of more interest. There are still many other measures not included in this comparison analysis. Their performance can also be evaluated using the proposed simulation experiments or inferred by exploring the connections of their defined formulas with those discussed here.

## Supporting information

S1 Table Autism dataset. This is a tab-delimited file with relative abundances summarized at the genus level.
(CSV)

S1 File R code. Functions that generate the compositional clusters in simulation experiments 1, 2, and 3, as described in detail in the Results section.
(R) Click here for additional data file.
